# Genome sequences of two *Arthrobacter* phages isolated from soil

**DOI:** 10.1128/mra.01300-23

**Published:** 2024-04-23

**Authors:** Dana Marchesin-Cupello, Gabriel Roscales, Paula Cobeta, Ruben Chaboy-Cansado, Daniel Aguirre de Cárcer, Alberto Rastrojo

**Affiliations:** 1 Department of Biology, Microbial and Environmental Genomics Group, Universidad Autónoma de Madrid, Madrid, Spain; Portland State University, Portland, Oregon, USA

**Keywords:** phages, genome assembly, temperate, *Arthrobacter*

## Abstract

The isolation and characterization of additional phages is crucial for adding reliable viral sequences with relevant biological information to viral databases. In this study, we present the complete genomes of two *Arthrobacter* phages obtained from different soil samples.

## ANNOUNCEMENT

A large fraction of viral metagenomic sequences remain poorly characterized, leading to a continuous increase in the so-called viral dark matter (1). The isolation and characterization of novel viruses is important for populating databases with reliable viral sequences that can enhance metagenomics studies. Here, we present the genome sequence of two phages recovered from soil infecting *Arthrobacter* sp. B10-11 isolated from the tomato rhizosphere (2).

**Fig 1:**
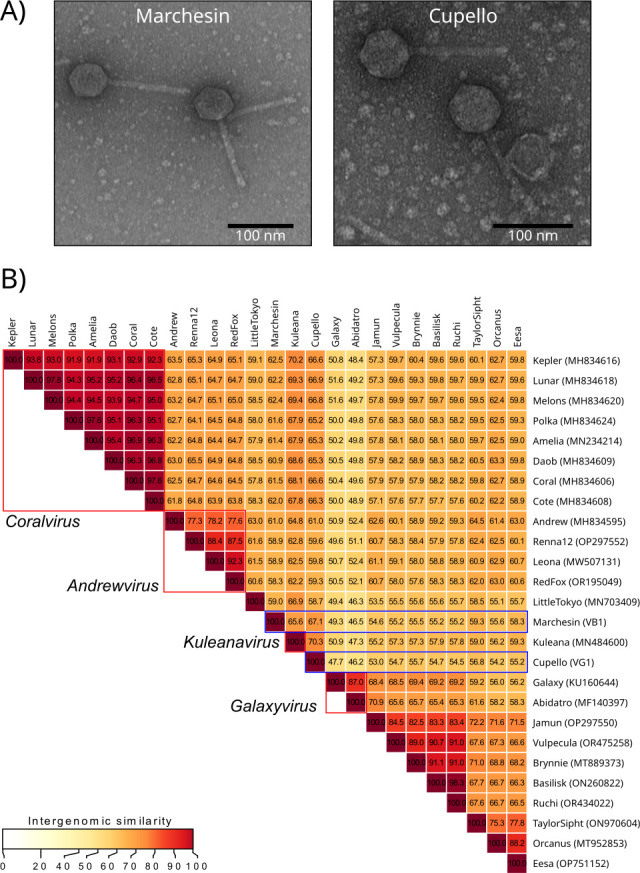
**(A**) Transmission electron microscopy micrograph of phages Marchesin (left panel) and Cupello (right panel) stained with 2% uranyl acetate. Images were obtained with a JEM 1010 (Jeol) microscope with an acceleration voltage of 80 kV coupled with a digital camera (TemCAM F416). (**B**) Intergenomic similarity analysis Virus Intergenomic Distance Calculator (VIRIDIC) performed among all *Arthrobacter* phages belonging to the AS cluster (PhagesDB). Red squares indicate defined viral genera recognized by The International Committee on Taxonomy of Viruses and blue squares indicate the two new sequenced phages presented in this work.

Phages were isolated from two fresh soil samples: one from a backyard garden in Madrid (Spain), and the other from a crop field in Badajoz (Spain). Briefly, 20 g of soil was shaken with 20 mL of phage buffer (50 mM Tris-HCl, 2 mM MgCl_2_, pH = 8) for 10 minutes and centrifuged at 4,000 × *g* for 20 minutes. Then, 0.22 µm filtered supernatants were concentrated using 100 kDa centrifugal units. The viral concentrates were then immediately used for double-layer agar assays (in Reasoner's 2A [R2A] media) using B10-11 as the host. After 24–72 h of incubation at 28°C, plaques were picked and re-isolated three times by using a double-layer agar assay. Two of the phages, named Marchesin and Cupello according to PhagesDB naming recommendations (3), were selected based on their plaque size and emergence time (3 mm/24 h or 2 mm/72 h, respectively), showing both a siphovirus-like morphology under electron microscopy (Fig. 1A). Phages were amplified using double agar layer method. Phages were purified from the upper layers by shaking in 20 mL of phage buffer that were centrifuged and concentrated as described above for soil samples and then treated with benzonase (250 U) before DNA extraction using the GeneJET Viral DNA and RNA Purification Kit (Thermo Fisher). DNA was randomly amplified using the GenomiPhi V3 DNA Amplification Kit (Cytiva). Illumina DNA prep kit was used for library preparation before sequencing on a MiSeq device (Illumina) using V2 chemistry.

A total of 931,372 and 679,740 pairs of reads (2 × 151 bp) were obtained for the Marchesin and Cupello libraries, respectively, that were quality filtered using Trimmomatic (v.0.39, minimum_quality = 30 and minimum_read_length = 100) (4) and digitally normalized using BBnorm (v.38.96, target_coverage = 100, sourceforge.net/projects/bbmap/). Then, SPAdes (v.3.14, using *--*careful option) (5) was used for genome assembly, resulting in a 36,722 bp genome for the Marchesin isolate and a 38,789 bp genome for Cupello (66.2%/66.95% GC and 254×/253× coverage, respectively). Both genomes were found to be almost complete based on CheckV analysis (v.1.0.1) (6) (99.98% and 100% completeness, respectively). Gene annotation was performed using Pharokka (v.1.3.2) (7), obtaining 75 and 76 coding genes for Marchesin and Cupello, respectively. Both genomes only showed nucleotide similarity (using FastANI v.1.34) (8) with other phages infecting *Arthrobacter* from cluster AS, as defined by PhagesDB. All phages from the AS cluster had a siphovirus-like morphology, an average genome size of 38 kb, and a temperate lifestyle. Accordingly, both isolates contained predicted integrase genes and were classified as temperate by PHACTS (v.1.8) (9). A more comprehensive taxonomic analysis, using VIRIDIC (v.1.1) (10), showed the closest relative was Kuleana, with 70% similarity with Cupello, suggesting that Cupello likely belongs to the *Kuleanavirus* genus, and with 65.6% similarity with Marchesin, which could potentially be considered as belonging to a new genus (Fig. 1B).

## Data Availability

The genome sequence has been deposited at NCBI under BioProject PRJNA1024081 and BioSamples SAMN38082476 and SAMN38082406. Trimmed Illumina reads can be found under SRA run accession numbers SRR26678894 and SRR26678895. Assembled genomes were deposited at GenBank with accession numbers OR777151 and OR777152.
